# Anal Canal Duplication in an 11-Year-Old-Child

**DOI:** 10.1155/2013/503691

**Published:** 2013-09-15

**Authors:** S. Van Biervliet, E. Maris, S. Vande Velde, D. Vande Putte, V. Meerschaut, N. Herregods, R. De Bruyne, M. Van Winckel, K. Van Renterghem

**Affiliations:** ^1^Departement of Pediatric Gastro-Enterology, Ghent University Hospital, De Pintelaan 185, 9000 Ghent, Belgium; ^2^Departement of Pediatric Surgery, Ghent University Hospital, De Pintelaan 185, 9000 Ghent, Belgium; ^3^Departement of Pediatric Radiology, Ghent University Hospital, De Pintelaan 185, 9000 Ghent, Belgium

## Abstract

Anal canal duplication (ACD) is the least frequent digestive duplication. Symptoms are often absent but tend to increase with age. Recognition is, however, important as almost half of the patients with ACD have concomitant malformations. We present the clinical history of an eleven-year-old girl with ACD followed by a review of symptoms, diagnosis, treatment, and prognosis based on all the reported cases in English literature.

## 1. Case Report

**Figure 1:**
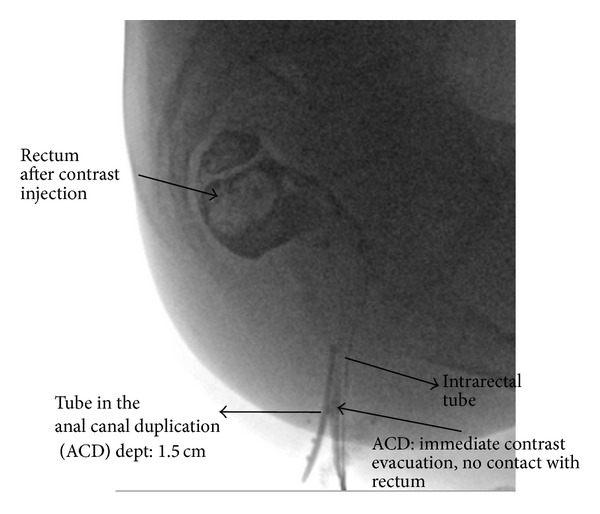
Fistulography revealing a blind ending tubular structure.

An eleven-year-old foster child was referred to the paediatric gastroenterology department because of an extra perianal orifice. The patient complained of anal pruritus. Previous treatment with mebendazole because of the suspicion of oxyuriasis had no effect. Physical examination revealed an extra orifice, in the midline posterior to the anus. Rectal palpation was normal. The anal canal appeared normal, with normal anal reflexes. This extra orifice had been observed at birth, with an expectative management advised in her native country. Cardiac ultrasound was normal. Magnetic resonance imaging (MRI) revealed a normal sacrum and coccyx but could not demonstrate the extra orifice or fistula. The genitourinary system, as evaluated in MRI, was normal. Fistulography (Figure 1) showed a 1.5 cm blind-ending fistula, not communicating with the rectum.

The patient and her parents were counselled about the diagnosis of ACD and the possible complications: inflammation and malignancy. Nevertheless they refused surgical mucosal stripping.

## 2. Discussion

ACD is the least frequent digestive duplication. Clinically, it presents itself as an extra perineal orifice located just behind the anus. Clinically, it is difficult to differentiate ACD from a rectal or anal fistula, however, in noncomplicated ACD inflammation will be absent. Only histology gives diagnostic certainty describing 3 characteristics of ACD: squamous epithelium in the caudal end, transitional epithelium in the cranial end and smooth-muscle cells in the wall of the canal [1, 2]. It is most frequently a tubular (90%) anomaly without communication to the rectum. In 10% of cases, the lesion is cystic [3]. We found only 55 patients (including our patient) with ACD in English literature (Table 1). Females comprise up to 89% of the patients with ACD (Table 1).

Two hypotheses concerning the origin of anal canal duplication are suggested in literature.

Choi and Park postulate it as a consequence of recanalization of a cloacal membrane excess in late embryonic life [4]. Hamada et al. suggest a duplication of the dorsal cloaca in an early developmental stage [5]. 

Half of the patients with ACD are asymptomatic. Parents or caregivers notice a perianal orifice posterior to the anus. Mild symptoms such as anal pain, pruritus, mucous discharge and constipation are present in one third of the patients. Perineal abscess or inflammation can, however, be the presenting complication of ACD. Although ACD is present at birth, it can easily be overlooked resulting in a widely varying age at presentation (Table 1). Diagnosis at a later age is more often associated with complications [3]. In the reported cases, there is a significant age difference according to the symptom severity (*P* < 0.03) with a median age in the asymptomatic reported patient of 0.8 y (minimum and maximum 0–9 y), in the patients with mild symptoms 4 y (0.1–16 y) and in the patient with complications 6.5 y (0.1–45 y). Inflammation, due to the presence of mucosal glands, infection, abscess formation, and subsequent sepsis are the immediate risks. On the long term, Dukes and Galvin reported malignancy in 8 of 10 adult patients of what they believed to be ectopic tracks of congenital origin [6]. Almost all articles on ACD use this old reference to warn about the risk of malignancy. However, the patients described by Dukes and Galvin are 90% males and suffer from multiple fistulas as can be seen on the clinical pictures of the paper whereas ACD patients in more recent publications are in 89% of cases female with only one orifice. As the wall of the ACD consists of squamous and transitional epithelium, unremarked degeneration of the mucosa in this duplication remains possible. 

Clinical suspicion and characteristics can lead to a tentative diagnosis of ACD. Imaging studies give extra information on the extent of the lesion and concomitant anomalies. MRI of the pelvis and presacral area gives a detailed view of the region. In neonates, however, ultrasound examination is preferred as they require general anaesthesia for MRI. Associated malformations are described in 35% (Table 1), including genitourinary malformations (ureteric duplication, external genitalia anomalies), congenital heart defects, cleft palate presacral mass (teratoma, dermoid cyst), sacral dysgenesis, and other anorectal malformations. 

It is advised to treat even asymptomatic ACD with surgery to prevent malignancy and infectious complications and to get diagnostic certainty with the histological examination of the excised material. Different approaches are suggested in literature. The majority of patients received an ACD removal via perianal or posterior sagittal approach. Mucosal stripping of the ACD is a new, less invasive approach most frequently used when the ACD is located very close to the anal canal. Surgical repair is associated with good prognosis and minor surgical sequelae. Up to now only one patient suffers from sphincter insufficiency [4]. 

## 3. Conclusion

Anal canal duplication is an extremely rare congenital anomaly of the digestive tract. A posterior perianal orifice, particularly in female patients, sometimes accompanied by aspecific symptoms should raise the suspicion of anal canal duplication. Clinical suspicion can be elaborated by imaging studies visualising the ACD and associated anomalies. Surgical removal, before the age of 1, is advocated to prevent complications. Histology gives confirmation of this anomaly.

## Figures and Tables

**Table 1:** Summary of all reported anal canal duplication cases in English literature.

Reference	No.	Sex	Localization	Type	Symptoms	Age	Associated anomalies
Our patient	1	1 F	Post	1 tub	1 mild	11 y	None
Sinnya (2012) [7]	1	1 F	Post	1 tub	1 complication	15 y	Dysplastic coccyx
Lippert (2012) [8]	1	1 F	Post	1 cyst	1 complication	12 y	None
Narci (2010) [9]	2	2 F	Post	2 tub	2 asympt	5 y (1–9 y)	None
Koga (2010) [10]	10	10 F	Post	10 tub	3 asympt 6 mild 1 complication	6 m (24 d–4 y)	1 hypoplastic kidney 1 teratoma and thetered cord 2 anal stenosis
Carpentier (2009) [2]	2	1 F 1 M	Post	1 tub 1 cyst	1 mild 1 complication	2.5 m (2-3 m)	1 spina bifida occulta, thetered cord, hydronefrosis 1 none
Kratz (2008) [11]	1	1 F	Post	1 cyst	1 complication	16 y	None
Lisi (2006) [3]	12	11 F 1 M	Post	10 tub 1 cyst	6 asympt 4 mild 2 complication	17.8 m (0–60 m)	1 anorectal malformation 1 cleft lip, cleft palate, omphalocoele 1 presacral ependymoma 2 teratoma's
Tiryaki (2006) [12]	2	2 F	Post	2 tub	1 asympt 1 mild	7 y (7–7 y)	1 none 1 intrasacral meningocele
Choi (2003) [4]	6	6 F	Post	6 tub	6 asympt	4.5 m (3–9 m)	6 none
Ochiai (2002) [1]	1	1 F	Post	1 combined	1 mild	6 y	None
Jacquier (2001) [13]	6	6 F	Post	6 tub	5 asympt 1 mild	2.5 m (0 m–12 y)	1 sacral teratoma, lumbosacral meningomyelocoele 1 sacral teratoma 1 uteric duplication 1 malrotation
Ponson (2001) [14]	3	3 F	Post	3 tub	1 asympt 2 mild	23 m (10 m–4 y)	3 none
Hamada (1996) [5]	2	2 F	Post	2 tub	1 asympt 1 mild	3.5 y (7 m–6 y)	1 cleft lip 1 none
Tagart (1977) [15]	4	1 F 3 M	3 right side 1 post	3 tub 1 cyst	4 complication	29 y (11 m–45 y)	None
Aaronson (1970) [16]	1	1 F	Post	1 tub	1 asympt	3 m	1 anterior sacral meningocoele, covered anus

Total group numbers	55	49 F 5 M	52 post 3 right side	48 tub 1 combined 6 cyst	26 asympt 18 mild 11 complication	4.6 y (0–45 y)	20 associated anomalies
Total group percentage %		89% F 11% M	94.5% post 5.5% right side	87% tub 2% combined 11% cyst	47% asympt 33% mild 20% complications		36% associated anomalies

Overview of the reported cases in English literature (first author and year of publication between brackets) with the number (No.) of reported cases, the localization (post: posterior), type of lesion (tub: tubular, cyst: cystic, both combined), presenting symptoms (asympt: asymptomatic; mild: mild symptoms (pruritus, discharge, constipation, diarrhea, and limited pain); complications (inflammation, abscedation)), age mean age and range between brackets in days (d), months (m), or years (y), and number and type of associated anomalies.
